# Analysis of the diagnostic efficacy of the QuantiFERON-TB Gold In-Tube assay for preoperative differential diagnosis of spinal tuberculosis

**DOI:** 10.3389/fcimb.2022.983579

**Published:** 2022-09-20

**Authors:** Xiaojiang Hu, Hongqi Zhang, Yanbin Li, Guang Zhang, Bo Tang, Dongcheng Xu, Mingxing Tang, Chaofeng Guo, Shaohua Liu, Qile Gao

**Affiliations:** ^1^ Department of Spine Surgery and Orthopaedics, Xiangya Hospital, Central South University, Changsha, China; ^2^ National Clinical Research Center for Geriatric Disorders, Xiangya Hospital, Central South University, Changsha, China; ^3^ Department of Clinical Laboratory, Xiangya Hospital, Central South University, Changsha, China

**Keywords:** spinal tuberculosis, QuantiFERON-TB Gold In-Tube (QFT-GIT), QFT-GIT, interferon-gamma release tests, IGRAs. ROC curve, disease duration, T-SPOT.TB

## Abstract

**Background:**

Differential diagnosis of spinal tuberculosis is important for the clinical management of patients, especially in populations with spinal bone destruction. There are few effective tools for preoperative differential diagnosis in these populations. The QuantiFERON-TB Gold In-Tube (QFT-GIT) test has good sensitivity and specificity for the diagnosis of tuberculosis, but its efficacy in preoperative diagnosis of spinal tuberculosis has rarely been investigated.

**Method:**

A total of 123 consecutive patients with suspected spinal tuberculosis hospitalized from March 20, 2020, to April 10, 2022, were included, and the QFT-GIT test was performed on each patient. We retrospectively collected clinical data from these patients. A receiver operating characteristic (ROC) curve was plotted with the TB Ag-Nil values. The cutoff point was calculated from the ROC curve of 61 patients in the study cohort, and the diagnostic validity of the cutoff point was verified in a new cohort of 62 patients. The correlations between TB Ag-Nil values and other clinical characteristics of the patients were analyzed.

**Results:**

Of the 123 patients included in the study, 51 had confirmed tuberculosis, and 72 had non-tuberculosis disease (AUC=0.866, 95% CI: 0.798-0.933, P<0.0001). In patients with spinal tuberculosis, the QFT-GIT test sensitivity was 92.16% (95% CI: 80.25%-97.46%), and the specificity was 67.14% (95% CI: 54.77%-77.62%). The accuracy of diagnostic tests in the validation cohort increased from 77.42% to 80.65% when a new cutoff point was selected (1.58 IU/mL) from the ROC curve of the study cohort. The TB Ag-Nil values in tuberculosis patients were correlated with the duration of the patients’ disease (r=0.4148, P=0.0025).

**Conclusion:**

The QFT-GIT test is an important test for preoperative differential diagnosis of spinal tuberculosis with high sensitivity but low specificity. The diagnostic efficacy of the QFT-GIT test can be significantly improved *via* application of a new threshold (1.58 IU/mL), and the intensity of the QFT-GIT test findings in spinal tuberculosis may be related to the duration of a patient’s disease.

## 1 Introduction

Spinal tuberculosis is a secondary infection caused by *Mycobacterium tuberculosis* and accounts for approximately 50% of all bone tuberculosis cases. *M.tuberculosis* can be transmitted to the spine by blood and lymphatic transmission from pulmonary tuberculosis, but it is also commonly seen in the spread of tuberculosis of the bladder, kidney, etc. More than 100,000 people are diagnosed with spinal tuberculosis each year worldwide, and China is a high-burden country for tuberculosis (Dunn and Ben Husien, 2018; Harding, 2020). The symptoms of spinal tuberculosis are often not obvious in the early stages and therefore often do not attract the attention of patients. Spinal tuberculosis can have serious consequences, such as kyphosis, spinal cord injury, and, in 10-30% of patients, even paraplegia. Therefore, early diagnosis and regular antituberculosis treatment are of great importance for improving the prognosis of patients with spinal tuberculosis (Go et al., 2012; Wang et al., 2020). Currently, spinal tuberculosis is often diagnosed on the basis of postoperative microbial culture (Perronne et al., 1994), the GeneXpert MTB/RIF system (Patel et al., 2020; Qi et al., 2022), and metagenomic next-generation sequencing (mNGS) (Shi et al., 2020; Sun et al., 2021). These diagnostic techniques require invasive tests or open surgery to obtain specimens, often delaying the patient’s confirmation of the diagnosis. The diagnostic efficacy of many preoperative tests is unsatisfactory: the CT, MRI, and other imaging techniques used are very similar to those used for pyogenic spinal infections, spinal brucellosis, and spinal tumors, and it is often difficult to distinguish these diseases by imaging (Kanna et al., 2019; Miyamoto and Akagi, 2019; Shroyer et al., 2021). While serologic indices such as white blood cell count (WBC), erythrocyte sedimentation rate (ESR), C-reactive protein (CRP), and procalcitonin (PCT) have been shown to play significant roles in differentiating active tuberculosis from non-tuberculosis, they lack specificity in differential diagnosis of spinal tuberculosis. Therefore, there is an urgent clinical need for a valuable diagnostic method for differential diagnosis of spinal tuberculosis in the preoperative period (Chen et al., 2016).

Currently, early diagnosis of *M. Tuberculosis* infection can be made using the tuberculin skin test (TST), which determines the status of tuberculosis *via* intradermal injection of purified protein derivatives from *M. Tuberculosis* strains. However, the TST has its limitations: age, smoking, diabetes, and BCG vaccination can cause false-positives in the TST and affect the determination of the results (Lu et al., 2021). In addition, interferon-gamma (IFN-γ) release assays (IGRAs) can be used to diagnose tuberculosis (Cho et al., 2010). IGRAs include common assays such as the T-SPOT.TB test, the QuantiFERON-TB Gold In-Tube (QFT-GIT) test, and the QuantiFERON-TB Gold Plus assay (Hamada et al., 2021). In China, a relatively widely used method is the T-SPOT.TB test, which assesses the presence of tuberculosis by stimulating T cells with the *M. Tuberculosis*-specific antigens ESAT-6 and CFP-10 and determining the numbers of spots obtained by the reaction. The QFT-GIT has demonstrated higher sensitivity than the T-SPOT.TB test in many studies (Harada et al., 2008). The QFT-GIT test is an immunological method for the diagnosis of tuberculosis that uses an enzyme-linked immunosorbent assay (ELISA) to detect the levels of IFN-γ produced upon stimulation with the tuberculosis-specific antigens ESAT-6, CFP-10, and TB7.7, which are specifically expressed by *M.tuberculosis* and all BCG strains and the majority of non-tubercle bacteria do not contain these antigens. The immune response to *M. Tuberculosis* is calculated by subtracting the background level from the stimulation level (TB Ag-Nil), which informs the diagnosis of tuberculosis (Shafeque et al., 2020)

The QFT-GIT test has been widely used in the clinical diagnosis of pulmonary tuberculosis (Lu et al., 2021) and has shown good sensitivity and specificity for this application, but its diagnostic efficacy for spinal tuberculosis is unclear (Harada et al., 2008). Unlike pulmonary tuberculosis, spinal tuberculosis is mainly caused by hematogenous dissemination of tuberculosis bacilli and needs to be clinically differentiated from pyogenic infection of the spine, brucellosis infection of the spine, and spinal tumors (Perronne et al., 1994). Studies have shown that diseases such as septic infections and cancers may also cause a nonspecific increase in INF-γ, resulting in false-positive results in the T-SPOT.TB and QFT-GIT tests (Choi et al., 2018). Therefore, the sensitivity and specificity of the QFT-GIT test in the diagnosis of spinal tuberculosis differs from that in the diagnosis of pulmonary tuberculosis, and a more appropriate diagnostic threshold is needed for differential diagnosis of spinal tuberculosis using the QFT-GIT test. In this study, we analyzed 123 patients with suspected spinal tuberculosis through a multicenter retrospective study. Based on the final diagnoses of the patients, we analyzed the sensitivity and specificity of the QFT-GIT test, plotted receiver operating characteristic (ROC) curves from the TB Ag-Nil values to find a suitable cutoff point for spinal tuberculosis, and analyzed the diagnostic efficacy of the adjusted cutoff point to improve the diagnostic efficacy of the QFT-GIT test in spinal tuberculosis. Finally, we correlated the TB Ag-Nil values of the QFT-GIT test with other clinical data of the patients to find a clinical indicator that may be correlated with the results of the QFT-GIT test in tuberculosis patients. We hope that this study will provide a relevant basis for the early diagnosis of patients with spinal tuberculosis.

## 2 Method

### 2.1 Study design

#### 2.1.1 Inclusion and exclusion criteria

A total of 123 patients with suspected spinal tuberculosis who were hospitalized at Xiangya Hospital, Xiangya Boai Hospital, Changsha First Hospital and Hunan Chest Hospital from March 20, 2020, to the present were selected. The inclusion criteria were as follows: 1. patients who were able to be diagnosed with tuberculosis or non-tuberculosis according to diagnostic criteria, and **2**. patients who were admitted for QFT-GIT testing. The exclusion criteria were as follows: 1. patients without QFT-GIT testing, 2. patients with autoimmune diseases, 3. patients with HIV infection, 4. patients with multiple infections, and 5. patients with severe underlying comorbid diseases. The Ethics Committee of Xiangya Hospital, Central South University, approved the implementation of this study(Ethical review number:201303232), and all enrolled subjects and their families gave written informed consent to the conduct of the study.

#### 2.1.2 Diagnostic criteria

According to the 2021 WHO guidelines for tuberculosis, the criteria for diagnosing spinal tuberculosis in this study were 1. a positive culture for *M. Tuberculosis*, 2. a positive Xpert result, 3. presence in an area with a high prevalence of tuberculosis meeting the appropriate histological features (containing at least one of the following three characteristics: caseous necrosis, granulomatous inflammation, and positive antacid staining) and effective antituberculosis treatment, and 4. detection of *M. Tuberculosis* genes in patient lesion specimens *via* mNGS (Sun et al., 2021). The criteria for diagnosing non-tuberculosis were as follows: 1. proof of other types of microbial infection by microbiological culture, PCR or mNGS; 2. pathological diagnosis of cancer or chronic septic inflammation (Coleman, 2006); and 3. no combination of pulmonary or other extrapulmonary tuberculosis.

### 2.2 QuantiFERON-TB Gold In-Tube test

A QFT-GIT test kit was purchased from QIAGEN (USA). The QFT-GIT test is used for *in vitro* qualitative detection of *M. Tuberculosis*-specific T-cell immunoreactivity in fresh anticoagulated human peripheral venous blood. After collecting a patient’s venous blood with an anticoagulation tube according to the kit instructions, the whole blood was added to three blood culture tubes, a blank control tube (Nil), a tuberculosis antigen tube (TB Ag) and a mitogen-positive control tube (Mitogen), each containing the *Mycobacterium tuberculosis*-specific antigens ESAT-6, CFP-10 and TB7.7 (p4). The whole blood was incubated in blood culture tubes at 37°C for 16-24 hours. Subsequently, the blood culture tubes were centrifuged, the plasma was collected, and the IFN-γ (IU/mL) content was determined by ELISA. The IFN-γ content of the TB Ag tube was subtracted from that of the Nil control tube and was determined to be positive if TB Ag-Nil ≥ 0.35 IU/mL. Due to the limited linear range of the ELISA standard curve, values greater than 10 IU/mL were defined as 10 IU/mL (Nikitina et al., 2016). The Mitogen and Nil wells were used for quality control of the results.

### 2.3 Statistical analysis

The sample data were statistically analyzed using SPSS 20.0 software and GraphPad Prism 9. The gender compositions and the proportions of patients with other comorbid diseases in the two groups were compared by chi-square test. The patients’ ages, BMI values, and WBC values conforming to a normal distribution are described using the mean ± standard deviation and were compared between groups *via* independent-sample t test. The duration of patients’ disease and the variables with nonnormal distributions such as patient course and C-reactive protein(CRP), erythrocyte sedimentation rate(ESR), and TB Ag-Nil values are described using the median and percentiles and were compared between groups *via* nonparametric rank sum test. Correlation analysis was performed using Spearman’s rho test. The results of the tests are expressed as P values, and P < 0.05 indicated a significant difference between the two groups. The results of the diagnostic tests were calculated on the website http://vassarstats.net/clin1.html#return. All figures in this paper were drawn using GraphPad Prism 9.0.

## 3 Results

### 3.1 Patient characteristics

We retrospectively collected data on 123 patients with suspected preoperative spinal tuberculosis from March 20, 2020, to April 10, 2022, at Xiangya Hospital, Xiangya Boai Hospital, Changsha First Hospital and Hunan Chest Hospital and identified 51 patients with tuberculosis and 72 patients without tuberculosis according to our diagnostic criteria. We used all patients in the period from March 20, 2020, to April 1, 2021, as the study cohort and used the patients admitted from April 2, 2021, to April 10, 2022, as the validation cohort. The study cohort contained 28 patients with tuberculosis and 33 patients with non-tuberculosis disease; the validation cohort contained 23 patients with tuberculosis and 39 patients with non-tuberculosis disease. The general characteristics of the two groups of patients are shown in **Table 1**; there were no significant differences in any characteristics between the study cohort patients and the validation cohort patients.

**Table 1:** The clinical characteristics of recruited patients in two independent cohorts.

	Study cohort		P^#^	Validation cohort		P^&^	P^$^
	TB (28)	NTB (33)		TB (23)	NTB (39)	
Age, years	53.93 ± 16.51	56.67 ± 13.41	0.478	53.57 ± 16.28	53.33 ± 13.58	0.952	0.454
Sex, male, %	16 (57)	19 (58)	0.973	14 (61)	25 (64)	0.799	0.531
Course, days	105 (45,180)	60 (42,135)	0.382	90 (30,210)	60 (30,150)	0.542	0.345
BMI, kg/m^2	22.71 ± 3.06	22.72 ± 3.72	0.989	21.51 ± 4.26	22.06 ± 3.99	0.689	0.256
Diabetes	1	4	0.363	0	5	0.148	1.000
Hypohepatia	2	3	1.000	3	2	0.350	1,000
Renal Insufficiency	0	1	1.000	0	1	1.000	1.000
Cancer	0	3	0.243	0	10	0.010	0.075
pulmonary tuberculosis	9	0	0.000	4	0	0.016	0.154
WBC, *10^9^/L	6.07 ± 2.26	6.73 ± 2.32	0.263	6.14 ± 2.20	7.08 ± 3.36	0.239	0.525
CRP, mg/L	11.34 (4.20,31.96)	17.95 (3.12,61.80)	0.733	25.58 (10.63,77.11)	13.83 (1.30,38.78)	0.133	0.326
ESR, mm/h	55.00 (29.25,81.00)	74.00 (33.00,112.50)	0.173	74.00 (63.00,107.00)	50.00 (35.00,104.00)	0.244	0.542
TB antigen positive, %	5 (18)	1 (3)	0.085	8 (35)	0 (0)	0.000	0.592
QFT-GIT positive, %	27 (96)	12 (36)	0.000	20 (87)	11 (28)	0.000	0.119

TB, spinal tuberculosis; NTB, non-tuberculosis;

^#^Comparisons were conducted between TB and NTB in study cohort.

^&^Comparisons were conducted between TB and NTB in validation cohort.

^$^Comparisons were conducted between the study cohort and validation cohort.

### 3.2 QFT-GIT test diagnostic efficacy

**Figure 1:**
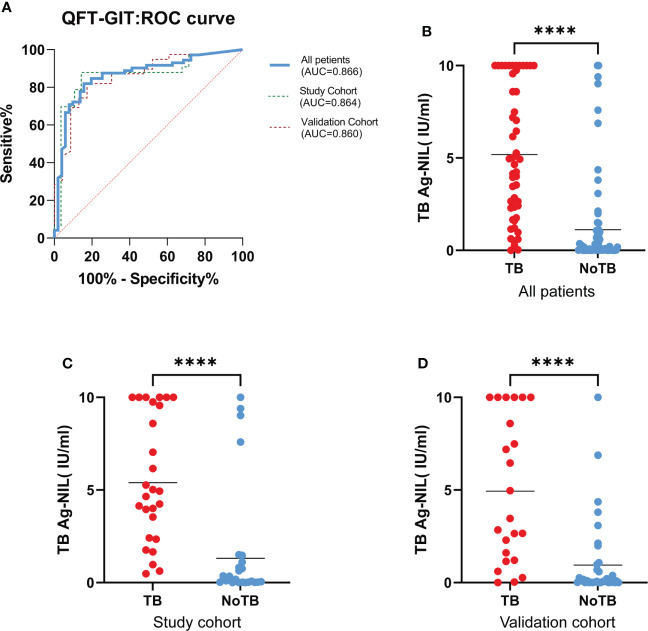
QFT-GIT diagnostic effectiveness. **(A)**. ROC curve by final diagnosis and TB Ag-Nil values in all 123 patients, study cohort and validation cohort; **(B, C, D)**. TB Ag-Nil values in the TB group than in patients in the NTB group, both in all patients, in the study cohort and in the validation. (**** means P ≤ 0.0001).

We plotted a ROC curve according to the final diagnosis and TB Ag-Nil values in 123 patients (**Figure 1A**) and found that the QFT-GIT test showed good diagnostic efficacy with an area under the curve (AUC)=0.866 (95% CI: 0.798-0.933, P<0.0001) in patients with spinal tuberculosis. The specific results are shown in **Table 2**. The sensitivity of the QFT-GIT test was 92.16% (95% CI: 80.25%-97.46%), the specificity was 67.14% (95% CI: 54.77%-77.62%), the positive predictive value was 67.14% (95% CI: 54.77%-77.62%), the negative predictive value was 92.45% (95% CI. 80.93%-97.55%), the positive likelihood ratio was 2.885 (95% CI: 2.040-4.080), the negative likelihood ratio was 0.115 (95% CI: 0.044-0.299), and the accuracy of the diagnostic test was 78.05%. We divided the 123 patients into a learning cohort and a validation cohort according to time, and the results and ROC curves of the respective diagnostic tests for both cohorts are also shown in **Table 2** and **Figure 1**. We observed significantly higher TB Ag-Nil values in the tuberculosis group than in the NTB group for all patients and for the two cohorts (learning and validation) (**Figures 1B–D**).

**Table 2:** Diagnosis performance of the QFT-GIT and adjusted QFT-GIT.

	AUC (95%CI)	Sensitive (95%CI)	Specificity (95%CI)	PPV (95%CI)	NPV (95%CI)	PLR (95%CI)	NLR (95%CI)	Accuracy
All patients (n=123)	0.866 (0.798-0.933)	92.16% (80.25%-97.46%)	68.06% (55.89%-78.28%)	67.14% (54.77%-77.62%)	92.45% (80.93%-97.55%)	2.885 (2.040-4.080)	0.115 (0.044-0.299)	78.05%
Study cohort (n=61)	0.864 (0.761-0.966)	96.43% (79.76%-99.81%)	63.64% (45.14%-79.04%)	69.23% (52.27%-82.45%)	95.45% (75.12%-99.76%)	2.652 (1.679-4.188)	0.056 (0.008-0.398)	78.69%
Validation cohort (n=62)	0.860 (0.764-0.955)	86.96% (65.33%-96.57%)	71.79% (54.90%-84.45%)	64.52% (45.38%-80.17%)	90.32% (73.10%-97.47%)	3.083 (1.824-5.212)	0.182 (0.062-0.532)	77.42%
Validation cohort (Based on adjusted cutoff point*)	0.860 (0.764-0.955)	73.91% (51.31%-88.92%)	84.62% (68.79%-93.59)	73.91% (51.31%-88.92%)	84.62% (68.79%-93.59%)	4.804 (2.213-10.428)	0.308 (0.154-0.619)	80.65%

TB, spinal tuberculosis; NTB, non-tuberculosis; AUC, the area under the curve; PPV, positive predictive value; NPV, negative predictive value; PLR, positive likelihood ratio; NLR, negative likelihood ratio; CI, confidence interval.

*adjusted cutoff point=1.58IU/mL

### 3.3 Diagnostic efficacy based on an adjusted cutoff point

We found a new cutoff point (1.58 IU/mL) from the ROC curve of 61 patients in the study cohort and determined whether the QFT-GIT test result was negative or positive based on the adjusted cutoff point in 62 patients in the validation cohort. The results of the diagnostic test are shown in **Table 2**. The sensitivity was reduced from 86.96% (95% CI: 65.33% -96.57%) -96.57%) to 73.91% (95% CI: 51.31%-88.92%); however, at the same time, the specificity increased from 71.79% (95% CI: 54.90%-84.45%) to 84.62% (95% CI: 68.79%-93.59). Using the adjusted cutoff value, the accuracy of the diagnostic test increased from 77.42% to 80.65% (**Table 2**).

### 3.4 Correlation analysis between the QFT-GIT test result and other indications

**Figure 2:**
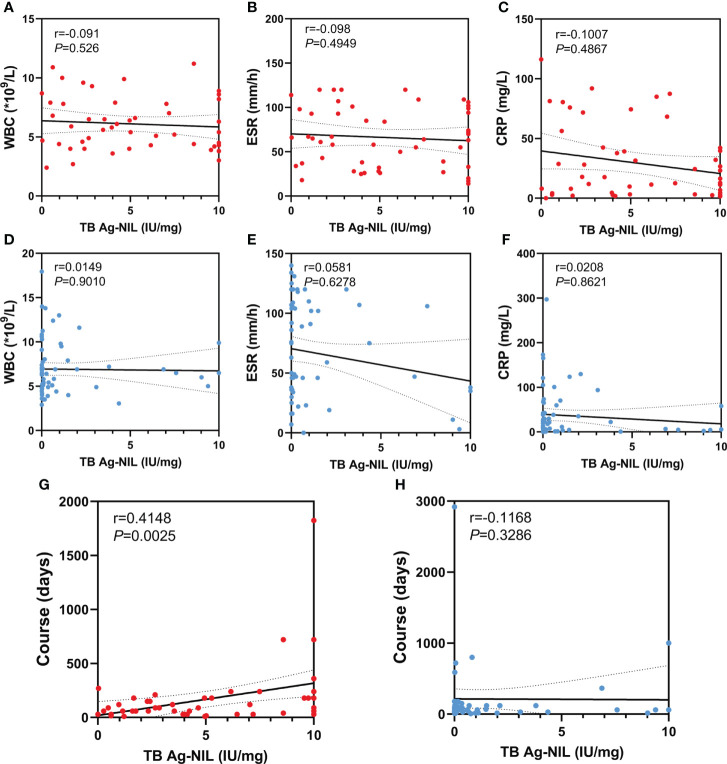
Correlation analysis between QFT-GIT and other indication: The red dots represent TB patients and the blue dots represent NTB patients. **(A, D)** Correlation analysis between TB Ag-Nil and WBC in TB patients or NTB patients; **(B, E)** Correlation analysis between TB Ag-Nil and ESR in TB patients or NTB patients; **(C, F)** Correlation analysis between TB Ag-Nil and CRP in TB patients or NTB patients; **(G, H)** Correlation analysis between TB Ag-Nil and course in TB patients or NTB patients;.

We analyzed the correlations between TB Ag-Nil and WBC, ESR, CRP and disease duration in a sample of 51 tuberculosis patients and 72 non-tuberculosis patients, respectively, by Spearman’s rho test. We found no correlations between TB Ag-Nil values and patients’ WBC, ESR and CRP in either the tuberculosis or non-tuberculosis groups (**Figures 2A–F**). Notably, our results showed a significant correlation between TB Ag-Nil and the duration of disease in tuberculosis patients (r=0.4148, P=0.0025) (**Figure 2G**) but not in non-tuberculosis patients (r=0.1168, P=0.3286) (**Figure 2H**).

## 4 Discussion

In this study, we enrolled 51 patients with tuberculosis and 72 patients with non-tuberculosis disease and assigned them to either the study cohort or the validation cohort according to the time of admission. There were no significant differences in age, sex, BMI, or duration of disease between the two cohorts. Nine patients in the non-tuberculosis group had a combined or final diagnosis of spinal tumor; notably, differential diagnosis between spinal tumors and spinal tuberculosis *via* imaging is sometimes difficult (Du et al., 2021). All these patients presented with vertebral bone destruction, and some spinal tumors were even visible on imaging as features consistent with spinal tuberculosis, such as abscesses of the psoas major muscle. Tuberculosis of the spine is often secondary to tuberculosis of other sites, most often pulmonary tuberculosis (Dunn and Ben Husien, 2018). In addition, patients with combined pulmonary or extrapulmonary tuberculosis were excluded from the non-tuberculosis group to avoid effects of these conditions on the QFT-GIT test. Our study also confirmed the inability to differentiate spinal tuberculosis from other diseases on the basis of ESR, CRP, and WBC. For comparison of these indicators between the two groups of patients, a nonparametric test was used, because the data for ESR and CRP did not conform to a normal distribution; in contrast, an independent-sample t test was used to analyze the differences in WBC between the two groups. As another challenge to differential diagnosis of spinal tuberculosis, septic and brucellosis infections are infectious diseases like tuberculosis, so these diseases also lead to increases in serum infection indicators (Ghassibi et al., 2021). Tumors can also sometimes cause the release of inflammatory substances, leading to increases in serum inflammatory marker levels in patients (Allin and Nordestgaard, 2011). Several of the above serologic indicators are not tuberculosis specific, and we did not see differences in these serologic indicators between patients with spinal tuberculosis and patients with non-tuberculosis disease in our data; therefore, early diagnosis of spinal tuberculosis by conventional inflammatory indicators is very difficult.

Our results showed that the QFT-GIT test showed high sensitivity (92.16%) in the diagnosis of spinal tuberculosis, and a high sensitivity can reduce underdiagnosis of spinal tuberculosis. However, the specificity of the QFT-GIT test was only 68.06%. The relatively low specificity may have been related to pyogenic infections of the spine caused by other pathogens, which can be caused by a variety of nonspecific microorganisms; certain nontuberculous mycobacterial infections, such as *Mycobacterium kansasii* (Johnston et al., 2017), *Mycobacterium szulgai* (Weng et al., 2020) and *Mycobacterium marinum* (Arend et al., 2002), may also cause positive test results in patients. The results of tests with low specificity can mislead physicians, causing them to use antituberculosis therapies for patients without tuberculosis. This has led to the treatment of misdiagnosed patients with unnecessary antituberculosis drugs with considerable adverse effects, including hepatotoxicity, optic nerve damage, and hearing loss (Yew et al., 2018; Ghassibi et al., 2021). In addition, incorrect antituberculosis treatment may lead to the emergence of drug-resistant bacteria (Nagel et al., 2017). Targeted anti-infective therapies can significantly shorten the course of the disease and improve the prognosis of patients (Kim et al., 2012).

To improve the specificity of the diagnostic test, we attempted to plot a ROC curve (AUC=0.866) with the continuous variable TB Ag-Nil, and we found that the QFT-GIT test showed good efficacy in the diagnosis of spinal tuberculosis. We further found a more appropriate cutoff point (1.58 IU/mL) in the study cohort for the diagnosis of spinal tuberculosis. We used this new cutoff point as a diagnostic criterion, specifying ≥1.58 IU/mL as positive in the QFT-GIT test. We reclassified the diagnostic test results in the validation cohort as being different from those in the study cohort and found that the QFT-GIT test showed better accuracy for the diagnosis of spinal tuberculosis with this new cutoff. The sensitivity was reduced to a certain degree, but the specificity of the diagnosis was improved.

We analyzed the correlations between TB Ag-Nil and WBC, ESR, CRP and disease duration in 51 tuberculosis patients and 72 non-tuberculosis patients in our sample and found that TB Ag-Nil was significantly positively correlated with patient course in the tuberculosis group. Previous studies have also shown that the intensity of the QFT-GIT test result is not related to the severity of tuberculosis but may be related to the activity of tuberculosis (Nikitina et al., 2016). The relationship between the intensity of the QFT-GIT test result and the duration of a patient’s disease has rarely been reported; however, a study of the intensity of the QFT-GIT test results at different periods of tuberculosis could not be performed because the present study was not designed as a follow-up investigation. This topic may deserve further study.

Clinical studies related to the QFT-GIT test have primarily focused on pulmonary tuberculosis, and a Japanese study showed that the sensitivity and specificity of the QFT-GIT test for the diagnosis of tuberculosis reached 92.6% and 98.8%, respectively (Harada et al., 2008). The QFT-GIT test shows high sensitivity and specificity in pulmonary tuberculosis. In 2018, Sungim Choi et al. (Choi et al., 2018) reported a clinical study on the QFT-GIT test in spinal tuberculosis in Korea. Their results of QFT-GIT test sensitivity (91%) and specificity (65%) were similar to our results showing both high sensitivity and low specificity. However, our study differed from theirs in several ways. First, our study included 51 patients with confirmed spinal tuberculosis, which is a relatively large sample size compared to those in other studies on osteoarticular tuberculosis and spinal tuberculosis. Second, our study was a multicenter study, and new patients will continue to be enrolled. Third, we plotted the ROC curve according to TB Ag-Nil values, divided patients into a study cohort and a validation cohort by admission time, found a new appropriate cutoff point from the ROC curve of the study cohort, and validated this new cutoff point in the validation cohort, demonstrating the improved diagnostic accuracy of this new cutoff point. Fourth, we found a correlation between TB Ag-Nil values and the duration of disease in patients with spinal tuberculosis *via* correlation analysis, providing a reference for preoperative differential diagnosis of spinal tuberculosis.

Notably, our study has some limitations. First, although the QFT-GIT examination was completed for all cases included in the study, a small amount of other biochemical examination data was missing for some cases due to refusal of some of the examinations, and some patients were missing BMI data because many patients with mobility problems could not cooperate to complete the height and weight measurements. Second, the present study was a preliminary study; we will correlate multiple indicators in later experiments, which may further improve the diagnostic efficacy. Based on the results of the current study, we will also conduct further prospective studies to improve the accuracy of preoperative differential diagnosis of spinal tuberculosis.

## 5 Conclusion

The QFT-GIT test is an important test for preoperative differential diagnosis of spinal tuberculosis with high sensitivity but low specificity. The diagnostic efficacy of the QFT-GIT test can be significantly improved *via* application of a new threshold (1.58 IU/mL), and the intensity of the QFT-GIT test findings in spinal tuberculosis may be related to the duration of a patient’s disease.

## Data availability statement

The original contributions presented in the study are included in the article/supplementary material. Further inquiries can be directed to the corresponding author.

## Author contributions

XH and QG designed research, performed research, analyzed data, and wrote the paper. HZ and YL developed the idea for the study. GZ, BT, DX collected the data. MT, CG and SL revised the paper. All authors contributed to the article and approved the submitted version.

## Funding

This study was supported by National Natural Science Foundation of China (No. 82072460,No. 82170901) and Natural Science Foundation of Hunan Province, China (No.2020JJ4892, No.2020JJ4908).

## Acknowledgments

I would particularly like to acknowledge the spine surgery of Xiangya Boai Hospital, Changsha First Hospital and Hunan Chest Hospital, for their wonderful collaboration and patient support.

## Conflict of interest

The authors declare that the research was conducted in the absence of any commercial or financial relationships that could be construed as a potential conflict of interest.

## Publisher’s note

All claims expressed in this article are solely those of the authors and do not necessarily represent those of their affiliated organizations, or those of the publisher, the editors and the reviewers. Any product that may be evaluated in this article, or claim that may be made by its manufacturer, is not guaranteed or endorsed by the publisher.
